# Cerebrospinal fluid markers to distinguish bacterial meningitis from cerebral malaria in children

**DOI:** 10.12688/wellcomeopenres.11958.2

**Published:** 2017-09-26

**Authors:** James M. Njunge, Ian N. Oyaro, Nelson K. Kibinge, Martin K. Rono, Symon M. Kariuki, Charles R. Newton, James A. Berkley, Evelyn N. Gitau

**Affiliations:** 1 KEMRI-Wellcome Trust Research Programme, Centre for Geographic Medicine Research Coast, Kilifi, Kenya; 2 University of Nairobi, Nairobi, Kenya; 3 Pwani University Health and Research Institute, Pwani University, Kilifi, Kenya; 4 Department of Psychiatry, Medical Sciences Division, University of Oxford, Oxford, OX3 7JX, UK; 5 Centre for Tropical Medicine and Global Health, University of Oxford, Oxford, OX3 7FZ, UK; 6 Alliance for Accelerating Excellence in Science in Africa (AESA), Nairobi, Kenya

**Keywords:** Biomarkers, Acute Bacterial Meningitis, Cerebral Malaria, CSF, proteomics, Myeloperoxidase, Lactotransferrin

## Abstract

**Background** **.** Few hospitals in high malaria endemic countries in Africa have the diagnostic capacity for clinically distinguishing acute bacterial meningitis (ABM) from cerebral malaria (CM). As a result, empirical use of antibiotics is necessary. A biochemical marker of ABM would facilitate precise clinical diagnosis and management of these infections and enable rational use of antibiotics.

**Methods.** We used label-free protein quantification by mass spectrometry to identify cerebrospinal fluid (CSF) markers that distinguish ABM (n=37) from CM (n=22) in Kenyan children. Fold change (FC) and false discovery rates (FDR) were used to identify differentially expressed proteins. Subsequently, potential biomarkers were assessed for their ability to discriminate between ABM and CM using receiver operating characteristic (ROC) curves.

**Results.** The host CSF proteome response to ABM ( *Haemophilus* *influenza* and *Streptococcus* *pneumoniae*) is significantly different to CM. Fifty two proteins were differentially expressed (FDR<0.01, Log FC≥2), of which 83% (43/52) were upregulated in ABM compared to CM. Myeloperoxidase and lactotransferrin were present in 37 (100%) and 36 (97%) of ABM cases, respectively, but absent in CM (n=22). Area under the ROC curve (AUC), sensitivity, and specificity were assessed for myeloperoxidase (1, 1, and 1; 95% CI, 1-1) and lactotransferrin (0.98, 0.97, and 1; 95% CI, 0.96-1).

**Conclusion.** Myeloperoxidase and lactotransferrin have a high potential to distinguish ABM from CM and thereby improve clinical management. Their validation requires a larger cohort of samples that includes other bacterial aetiologies of ABM.

## Introduction

Acute non-traumatic coma is an important cause of morbidity and mortality among paediatric hospital admissions in malaria endemic areas of Africa. This is commonly caused by acute bacterial meningitis (ABM) and cerebral malaria (CM), although viruses, fungi, and other infectious and non-infectious causes may occur. The clinical features associated with and used for diagnosis of ABM may overlap with those of CM. The World Health Organization defines CM as coma that persists >1 h after a seizure once hypoglycaemia is corrected with no other cause to explain the coma, and the presence of asexual parasites in peripheral blood ^1^. Abnormal retinoscopy is associated with cerebral parasite sequestration and increases the specificity of the diagnosis for CM ^2, 3^. Histidine-rich protein 2, a parasite protein used to estimate the total body parasite biomass, is also considered a potential marker that increases specificity for CM ^4, 5^, but does not exclude ABM. In practice, children with clinical signs and a positive malaria slide or rapid diagnostic test (RDT) are treated for malaria. However, in malaria-endemic regions, asymptomatic malaria parasitemia can be common and the presence of parasites may mean that a patient fulfils the diagnostic criteria for CM, when in fact another cause exists.

The diagnosis of bacterial meningitis is often more difficult. Cerebrospinal fluid (CSF) culture is the gold standard. Other surrogate markers include CSF pleocytosis with neutrophil predominance, low CSF glucose, and increased total CSF protein concentration. However, all these require laboratory facilities. CSF culture takes almost 48 hours, and although highly specific, has about 80% sensitivity, which is reduced when antibiotics have been given prior to sampling ^6, 7^. Thus, distinguishing patients with ABM can be difficult in malaria-endemic areas ^8– 10^. Consequences of failing to adequately treat ABM are increased risks of death and severe neuro-disability ^11^. On the other hand, unnecessary use of antibiotics risks escalating antimicrobial resistance ^12^. Therefore, a fast and reliable biochemical marker that could be developed into a point of care test with sufficient specificity and sensitivity would facilitate clinical diagnosis and appropriate management of CNS infections. Markers of the host response to CNS infection may offer the opportunity to distinguish infection aetiology to identify ABM.

Proteomic analysis allows quantitation of a large number of proteins present in biological fluids, such as plasma and CSF, providing an opportunity for unbiased discovery of biomarkers associated with clinical phenotypes ^13– 17^. Examples include aetiology-specific host response signatures that distinguish pneumococcal, meningococcal, and enteroviral meningitis ^18^; mortality risk in pneumococcal meningitis ^16^; and CM compared to other encephalopathies ^17^. The latter approach enhances proteome coverage, but generally precludes quantification of identified proteins.

In this study, we aimed to determine components of the CSF protein expression profiles of children with ABM that distinguish from those with CM with a high degree of specificity and sensitivity, which could be developed into point-of-care tests. 

## Methods

### Study participants

The study used archived CSF samples (n = 59) from paediatric admissions (2002–2011) at Kilifi County Hospital (Formerly Kilifi District Hospital), Kilifi, Kenya. All samples used in this study had been consented and approved for storage and research by the Kenya Medical Research Institute. Samples comprised two groups of children, based on clinical and laboratory findings. Acute bacterial meningitis (ABM; n=37) was defined as children who had a positive bacterial culture for CSF. Cerebral malaria (CM; n=22) was defined as children who had peripheral asexual malarial parasites >2500 parasites/μl on blood film ^19^, negative CSF cultures, CSF leukocyte count <10 cells/μl and no CSF biochemical feature of ABM. ABM was defined without respect to parasitemia, and therefore 5 children had *Plasmodium falciparum* coincidental infection.

### Sample preparation and LC-MS/MS analysis

Aliquots of 10 μl of CSF were denatured in 50 mM ammonium bicarbonate (Fluka) containing 8 M urea (Sigma). Proteins were reduced with 20 mM dithiothreitol (Sigma) at room temperature with shaking for 1 hour (h) and subsequently alkylated in the dark for 1 h with 65 mM iodoacetamide (Sigma). Excess iodoacetamide was quenched using 65 mM dithiothreitol. Urea present in the sample was dialyzed out with 50mM ammonium bicarbonate, using 3 kDa amicon filters (Millipore). Proteins were digested with trypsin (Thermo Scientific) overnight (16 hours) and peptides obtained were desalted using C18 Spin columns (Thermo Scientific), according to manufacturer’s instructions, dried in a Speedvac concentrator (Thermo Scientific), and re-suspended in 50 μl loading solvent (97.05% H _2_O, 2% acetonitrile, 0.05% formic acid). Peptides (5 μl) were loaded using a Dionex Ultimate 3000 nano-flow ultra-high-pressure liquid chromatography system (Thermo Scientific) on to a 75µm x 2 cm C18 trap column (Thermo Scientific) and separated on a 75µm x 25 cm C18 reverse-phase analytical column (Thermo Scientific). Elution was carried out with mobile phase B (80% acetonitrile with 0.1% formic acid) gradient (5 to 35 %) over 120 min. Peptides were measured using a Q Exactive Orbitrap mass spectrometer (Thermo Scientific) coupled to the chromatography system via a nano-electrospray ion source (Thermo Scientific). The ms^1 settings were: Resolution, 70000; AGC target, 3e6; scan range, 400–1800 m/z; while the ms^2 settings were: Resolution, 17500; AGC, 5e4; isolation window, 1.6 m/z. The top 15 most intense ions were selected for ms^2, which were subsequently excluded for the next 30 s.

### Data preparation

Mass spectrometer files ( .Raw files) were analysed by MaxQuant software version 1.5.3.30 ^20^ by searching against the human Uniprot FASTA database (downloaded February 2014) using the Andromeda search engine ^21^. Cysteine carbamidomethylation was set as a fixed modification and N-terminal acetylation and methionine oxidations as variable modifications. The false discovery rate (FDR) was set to 0.01 for both proteins and peptides with a minimum length of seven amino acids and was determined by searching a decoy database. A decoy FASTA database is generated from the target database, comprising sequences derived from the organism being studied, by switching the amino-carboxyl orientation of a protein’s amino acids to generate sequences that do not exist in nature, which are then concatenated with the target FASTA database ^22^. Enzyme specificity was set as C-terminal to arginine and lysine with trypsin as the protease. A maximum of two missed cleavages were allowed in the database search. Peptide identification was performed with an allowed initial precursor mass deviation of up to 7 ppm and an allowed fragment mass deviation of up to 20 ppm. The label free quantification (LFQ) algorithm in MaxQuant was used to obtain quantification intensity values.

### Statistical analysis

***a) Pre-processing and exploration.*** Study participants characteristics data was uploaded and analyzed in Stata version 13.1 and significance was tested using Wilcoxon rank-sum (Mann-Whitney) test for non-parametric variables, while two-sample t test with equal variances was used for parametric data. Chi square test was used for binary data.

Proteome data analysis was conducted in R version 3.3.2 and all samples were included in the analysis. We limited protein analysis to those identified and quantified in at least half of the samples in either ABM or CM. Proteins that were not detected in a sample were presumed to be on the lower limit of detection and their LFQ values were set at 0. Range and logarithmic normalization was performed to adjust protein quantities to a comparable scale. Unsupervised clustering using principal component analysis (PCA) and hierarchical clustering were applied to assess variation and determine group separation (i) among the ABM samples that comprised *Haemophilus influenza* (n = 12) and *Streptococcus pneumoniae* (n = 25) and (ii) between ABM and CM. Group separation among ABM samples was carried out to determine whether the host response is bacterial-specific or generic. To visualize protein clustering patterns, a heatmap was generated with the Pearson correlation coefficients as the distance metric.

***b) Biomarker extraction.*** Fold change (FC) and FDR were used to identify differentially expressed proteins. Selection of candidate biomarkers was performed through feature-importance assignment, based on variable importance, as implemented in the random forest (RF) algorithm version 4.6-12 ^23^. Here, recursive feature elimination (RFE) resulted in a reduced subset of proteins whose ability to distinguish between ABM and CM groups was evaluated using the mean decrease in accuracy (MDA) scores. The Boruta R package version 5.2.0 ^24^ is designed as a wrapper around RF facilitating RFE and MDA weight assignment. Subsequently, each protein was assessed for its ability to discriminate between ABM and CM by evaluating its receiver operating characteristic (ROC) curves. Potential biomarkers were thus identified from the differentially expressed proteins ranked according to the area under curve (AUC) and MDA scores.

## Results

### Characteristics of study participants

The study analysed 59 samples from two clinical groups, whose characteristics are presented in Table 1.

**Table 1.** Characteristics of study participants. Data are median (interquartile range), unless otherwise stated. Abbreviations: CSF, cerebrospinal fluid; iRBC, infected red blood cell; WBC, white blood cell; MUAC, mid-upper arm circumference.

Characteristic	Acute bacterial meningitis (n=37)	Cerebral malaria (n=22)	P
Age, months	35 (9 – 90)	30.5 (13 – 37)	0.61
Sex, male, n (%)	21 (56.8)	8 (36.4)	0.1
Parasite density, iRBCs ×10 ^3^/μL	0 (0 – 0)	230000 (100800 – 393600)	0.0001
CSF WBC count, cells/μL	3370 (288 – 5120)	2 (1 – 4)	0.0001
Total CSF protein, mg/dL	2.1 (1.3 – 2.49)	0.28 (0.22 – 0.37)	0.0001
Blood glucose, mg/dL	5.4 (4 – 7.6)	5.3 (3.2 – 7.7)	0.9
CSF glucose, mg/dL	0.6 (0.3 – 0.9)	3.1 (2.6 – 3.7)	0.0001
Ratio of CSF to blood glucose	0.1 (0.06 – 0.16)	0.69 (0.42 – 1)	0.0001
Outcome, dead, n (%)	13 (35)	0	0.001
MUAC, cm	13.95 (11.5 – 15.4)	13.95 (13.3 – 15.2)	0.3
Seizures, n (%)	9 (24)	10(46)	0.1

### CSF proteomes of ABM and CM differ significantly

**Figure 1.**
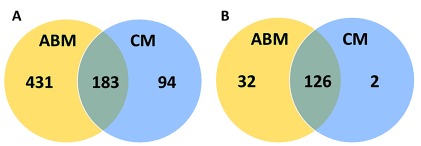
Cerebral spinal fluid (CSF) proteomes of acute bacterial meningitis (ABM) and cerebral malaria (CM) differ significantly. ( **A**) Distribution of total proteins quantified (n = 708) between ABM and CM. ( **B**) Distribution of proteins included in the biomarker analysis, where proteins had to be quantified in at least half of the samples in either ABM or CM. The CSF of ABM patients is characterized by a larger protein diversity compared to CM.

The LC-MS/MS analysis resulted in the quantification of a total of 708 non-redundant proteins, of which 183 proteins were commonly expressed in both ABM and CM ( Figure 1A). One hundred and sixty proteins were quantified in >50% within each group and were selected for further analysis ( Figure 1B). Of the 160 proteins, 32 proteins were not quantified in CM, while two proteins were not quantified in ABM, as shown in Figure 1B. Overall, ABM had a higher number of quantified proteins than CM. All quantified proteins and those included in subsequent analysis are listed in Table S1 and Table S2, respectively.

**Figure 2.**
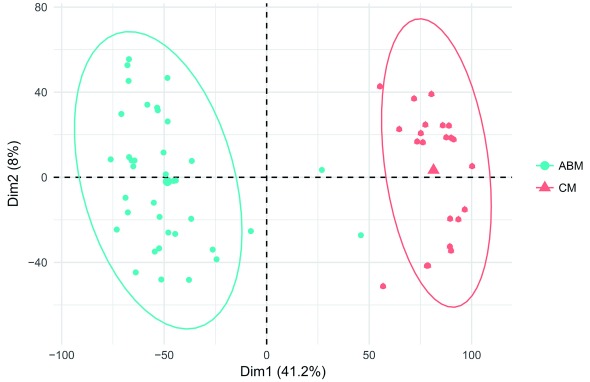
Host response acute bacterial meningitis (ABM) and cerebral malaria (CM) is pathogen specific. Unsupervised clustering using principal component analysis (PCA) was employed to determine clustering patterns of samples. The PCA score plot of the cerebral spinal fluid proteomes depicts clear group separation. Dimension (Dim) 1 of the PCA accounted for 41% of variation, while Dim 2 accounted for 8%.

Normalized protein LFQ values revealed clear sample separation into ABM and CM groups ( Figure 2). S100A8/S100A9 (calprotectin), lactotransferrin (LTF), myeloperoxidase (MPO), and myeloblastin (PRTN3) were the top five proteins driving the sample group separation observed in dimension 1 (data not visualised). The CM samples showed strong within-cluster connectivity, suggesting lower proteome variation compared to ABM samples, which showed greater cluster spread. The ABM group comprising Gram-negative *H. influenza* and Gram-positive *S. pneumoniae* did not exhibit any bacteria-specific clustering ( Figure S1).

**Figure 3.**
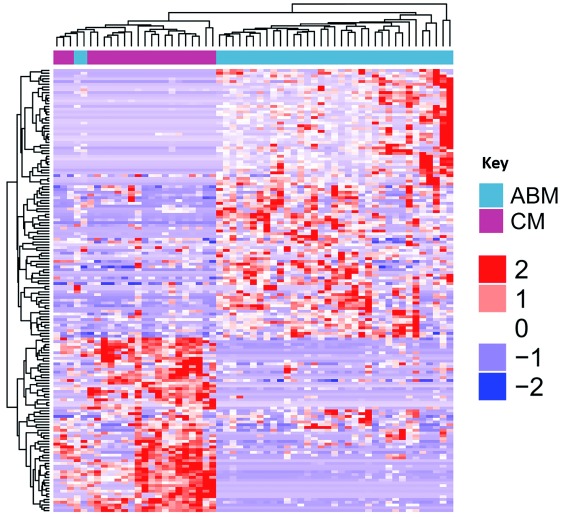
Heatmap demonstrating sample clustering based on protein expression profiles from acute bacterial meningitis (ABM) and cerebral malaria (CM). The heatmap was generated using hierarchical clustering based on protein expression levels calculated from normalized label free quantification values (2 and -2). The Pearson correlation coefficients were used as the distance metric. Rows represent individual proteins, while columns represents samples. Red indicates upregulation and blue indicates downregulation. Distinct sample clustering based on protein expression levels was observed with clear separation between the ABM and CM, except for two ABM samples that clustered with the CM.

In order to visualize clustering patterns of proteins based on normalized LFQ quantities, a heatmap with the rows, representing 160 proteins, and columns, representing 59 samples, was generated with the Pearson correlation coefficients as the distance metric ( Figure 3). The protein expression profiles distinguished the two groups ( Figure 3). It is notable that two ABM samples clustered with the CM group and this was similarly observed with the PCA analysis ( Figure 2 and Figure 3).

Analysis revealed that 52 proteins were differentially expressed (FDR<0.01, Log FC≥2) ( Table 2, Figure S2), of which 83% (43/52) were upregulated in ABM compared to CM ( Figure S2). Proteins including MPO, LTF, PRTN3, PFN1, LCN2, MMP8, MMP9, RETN, PGLYRP1, and S100A8/S100A9 were among the significantly expressed proteins in ABM, while SPARC, CNTN1, CHGB, and SPARCL1 were upregulated in CM ( Table 2).

**Table 2.** List of 52 quantified proteins that showed differential expression (FDR<0.01, and log FC≥2). FDR, false discovery rate; FC, fold change.

Protein ID	Protein name	Gene name	Log FC	*P* value	FDR
P05164-2	Myeloperoxidase	MPO	-31.7	8.32E-77	1.33E-74
P02788	Lactotransferrin	LTF	-32.8	5.05E-66	4.04E-64
U3KPS2	Myeloblastin	PRTN3	-30.8	9.89E-42	5.27E-40
P07737	Profilin-1	PFN1	-30.7	1.21E-37	4.85E-36
P29401	Transketolase	TKT	-30.6	1.23E-33	3.94E-32
P08670	Vimentin	VIM	-32	1.7E-28	4.54E-27
P13796	Plastin-2	LCP1	-32	1.41E-27	3.22E-26
P12814	Alpha-actinin-1	ACTN1	-30.5	1.32E-22	2.65E-21
P31146	Coronin-1A;Coronin	CORO1A	-29.6	3.36E-22	5.98E-21
P80188	Neutrophil gelatinase- associated lipocalin	LCN2	-30.6	2.67E-19	4.27E-18
P22894	Neutrophil collagenase	MMP8	-29.4	8.23E-19	1.2E-17
P09486	SPARC	SPARC	28.5	5.41E-18	7.21E-17
Q12860	Contactin-1	CNTN1	28.6	5.91E-18	7.27E-17
Q9HD89	Resistin	RETN	-29.9	1.11E-17	1.27E-16
P06744	Glucose-6-phosphate isomerase	GPI	-29.8	1.57E-17	1.68E-16
P14780	Matrix metalloproteinase-9	MMP9	-29.4	2.95E-17	2.95E-16
P80511	Protein S100-A12	S100A12	-31.2	1.8E-15	1.7E-14
P62937	Peptidyl-prolyl cis-trans isomerase A	PPIA	-30.1	3.59E-15	3.19E-14
P31949	Protein S100-A11	S100A11	-28.9	2.6E-14	2.19E-13
P26038	Moesin	MSN	-29.4	4.99E-13	3.99E-12
P11142	Heat shock cognate 71 kDa protein	HSPA8	-29.3	6.65E-13	5.07E-12
P06753-2	Tropomyosin alpha-3 chain	TPM3	-29.4	3.48E-12	2.53E-11
P60660-2	Myosin light polypeptide 6	MYL6	-28.9	5.27E-12	3.67E-11
P62158	Calmodulin	CALM1	-29.3	1.94E-11	1.3E-10
P08107	Heat shock 70 kDa protein 1A/1B	HSPA1A	-29.5	3.34E-11	2.14E-10
P04003	C4b-binding protein alpha chain	C4BPA	-28.5	1.32E-10	8.12E-10
O75594	Peptidoglycan recognition protein 1	PGLYRP1	-28.1	1.68E-10	9.93E-10
Q93079	Histone H2B type 1-H	HIST1H2BH	-30	2.73E-10	1.56E-09
Q01518	Adenylyl cyclase- associated protein 1	CAP1	-29.4	7.17E-10	3.95E-09
P04114	Apolipoprotein B-100; Apolipoprotein B-48	APOB	-28.3	1.07E-09	5.73E-09
P04083	Annexin A1	ANXA1	-29	3.69E-09	1.9E-08
P07900	Heat shock protein HSP 90-alpha	HSP90AA1	-28.9	4.54E-09	2.2E-08
P35579	Myosin-9	MYH9	-30.2	4.52E-09	2.2E-08
O43866	CD5 antigen-like	CD5L	-27.2	2.32E-08	1.07E-07
P00338	L-lactate dehydrogenase A chain	LDHA	-28.4	2.34E-08	1.07E-07
P02649	Apolipoprotein E	APOE	4.5	1.25E-05	5.56E-05
P36955	Pigment epithelium- derived factor	SERPINF1	3.5	1.45E-05	6.27E-05
P05090	Apolipoprotein D	APOD	2.2	2.54E-05	0.0001
P02766	Transthyretin	TTR	3.9	4.57E-05	0.0002
P06702	Protein S100-A9	S100A9	-9	5.58E-05	0.0002
P05109	Protein S100-A8	S100A8	-8.9	0.000161	0.0006
P23142-4	Fibulin-1	FBLN1	3.6	0.000219	0.0008
P02675	Fibrinogen beta chain	FGB	-4.9	0.000399	0.0014
P02679-2	Fibrinogen gamma chain	FGG	-4.7	0.000689	0.0025
P00738	Haptoglobin	HP	-5.2	0.000988	0.0032
P01034	Cystatin-C	CST3	3.7	0.001006	0.0032
P05060	Secretogranin-1	CHGB	7.9	0.000988	0.0032
P60709	Actin	ACTB	-5.9	0.000949	0.0032
P41222	Prostaglandin-H2 D-isomerase	PTGDS	4	0.001688	0.0052
P06733	Alpha-enolase	ENO1	-9	0.001814	0.0052
Q14515	SPARC-like protein 1	SPARCL1	8	0.00214	0.0063
P59666	Neutrophil defensin 3	DEFA3	-6.6	0.00245	0.0071

### MPO and LTF as the best biomarkers distinguishing ABM from CM

To identify top ranking biomarkers that distinguish ABM from CM, differentially expressed proteins were subjected to a feature-based weighting procedure where protein importance was assigned using the MDA scores. Higher scores imply increased ability of a protein to distinguish between ABM and CM ( Figure S3). The proteins selected by the algorithm as the most important biomarkers are listed on Table 3, where MPO, SPARCL1, and LTF rank as the top three proteins.

**Table 3.** Area under the curve (AUC), sensitivity (Sens.), specificity (Spec.), and mean decrease in accuracy (MDA) scores for the best performing biomarkers.

Biomarker	AUC (95% CI)	Sens.	Spec.	Classified correctly %	MDA
MPO	1.00 (1 to 1)	1	1	100	8.7
LTF	0.98 (0.96 to 1)	0.97	1	98	6.9
PRTN3	0.96 (0.91 to 1)	0.92	1	95	5.6
PFN1	0.96 (0.91 to 1)	0.92	1	95	5
TKT	0.95 (0.89 to 0.99)	0.89	1	93	4.1
VIM	0.91 (0.86 to 0.97)	0.83	1	90	2.98
LCP1	0.90 (0.84 to 0.97)	0.81	1	88	2.97
ACTN1	0.90 (0.84 to 0.97)	0.81	1	88	2.76
CORO1A	0.90 (0.84 to 0.97)	0.81	1	88	3.16
CSF Glucose (mg/dL) <2.4 ^*^	0.88 (0.80 to 0.99)	0.91	0.87	88	-
CSF WBC count, cells/μL >10 ^*^	0.95 (0.89 to 1)	1	0.90	97	-
Total CSF protein >0.54 ^*^	0.94 (0.87 to 1)	0.97	0.90	95	-

^*^indicates the best cut-off that achieved high sensitivity and specificity.

Consistently, there was an overlap of differentially expressed proteins and the top ranking biomarker proteins. Additionally, ROC curves were generated independently for each of the biomarkers and the AUC determined. The top ranking biomarkers were selected based on an AUC >0.9 and MDA scores above those of shadow proteins ( Table 3). However, among the biomarkers, MPO and LTF achieved high sensitivity (≥0.98) and specificity (1), depicting their predictive potential as biomarkers ( Table 3).

## Discussion

Clinical differentiation of ABM and CM is important as it dictates management and prognosis. A diagnostic test based on host proteins avoids heterogeneity in pathogen proteins between infecting bacterial species. We found significant proteome difference between ABM and CM, implying that the host responds differently to bacterial and *Plasmodial* infections. This is consistent with previous work indicating a differential host response in plasma and CSF of children with a diagnosis of CM compared to those with a malaria-slide-negative ABM ^17^. In a previous study, Gitau *et al*. reported differentially expressed proteins in plasma and CSF from children with CM, ABM, and nonspecific encephalopathies ^17^. The approach enhanced proteome coverage through pre-fractionation, but precluded relative quantification of proteins. Biomarker discovery has been enhanced by recent developments in mass spectrometry instrumentation ^25^ and advanced computational and bioinformatics algorithms ^20, 26^. In the present study, we performed shotgun proteomics and quantitative differences in host proteins in CSF.

ABM was characterized by a higher CSF total protein concentration and higher host protein diversity. This likely results from protein infiltration following breakdown in the blood brain barrier and secretion from host cells including infiltrating neutrophils. Whilst in CM the blood brain barrier is mildly impaired with few morphological changes ^27– 30^ and *Plasmodia* parasites are usually restricted to the vascular compartment rather than the meninges or the parenchyma of the brain, unless haemorrhage occurs.

The aetiology of ABM comprised Gram-negative *H. influenza* and Gram-positive *S. pneumoniae*. However, there was no species-specific clustering, implying that host responses to bacterial infections is largely generic. This homogeneity supports using host biomarkers for distinguishing meningitis.

A large proportion of the proteins that were not consistently quantified were excluded from the main analysis where biomarker mining followed criterion to include only proteins quantified in more than half of the samples from either of the two groups. This increased the reliability of a selected biomarker. Reducing the dataset in this way resulted in a substantial increase in proteins shared between the two groups, with ABM retaining a higher proportion of unique proteins compared to CM.

MPO and LTF were the most promising proteins for consideration as biomarkers based on their expression, sensitivity and specificity, as judged by the AUC and also ranked among the top biomarkers using RF. MPO contributes to innate host defences through microbial killing and is stored in large quantities in the azurophilic granules of neutrophils and released upon cell activation ^31, 32^. MPO and LTF are also part of neutrophil extracellular traps; fibrillar matrices comprised of chromatin and antimicrobial proteins, released by activated neutrophils ^33^. As a biomarker, MPO has previously been shown to be higher in the CSF of patients with infectious causes compared to those of non-infectious causes, and its levels have been shown to correlate well with neutrophil counts ^34^. MPO has already been developed as a diagnostic tool for cardiovascular disease risk stratification ^35^.

LTF is an antimicrobial polypeptide found in secondary granules of neutrophils and in human mucosal secretions, and plays a role in iron metabolism and inflammation ^36, 37^. LTF plays a bacteriostatic role in host defence due to its iron sequestering properties that inhibit bacterial proliferation. It is ineffective against bacteria able to acquire their iron from either LTF or transferrin ^38– 40^. Previously, LTF has been shown to be elevated in the CSF of patients with bacterial meningitis ^41– 44^. In a study in adults, CSF LTF showed diagnostic efficiency (AUC; 0.946, sensitivity; 96.6, specificity; 92.4) when distinguishing between bacterial and aseptic meningitis ^45^. Such findings point to LTF as a molecular marker for ABM. Currently, LTF can be assayed using ELISA, but has the potential to be developed as a diagnostic test. It is notable that both MPO and LTF performed better than surrogate markers in the CSF, including glucose, pleocytosis, and total protein. Determination of pleocytosis requires microscopy and couldn’t be a point-of-care test. Bed-side glucose rapid tests are not currently accurate enough to reliably distinguish ABM from CM.

In resource limited settings in Africa, point-of-care diagnostics could considerably help in diagnosis. In malarious areas, a rapid test sensitive and specific for ABM could help in identification and timely management of comatose children suffering from ABM even though they may have a positive malaria slide or RDT. Such an approach would contribute to antibiotic stewardship in the face of increasing resistance and improve resource use. The markers identified in this study await validation and development as point-of-care diagnostics.

Our study had several limitations. For ethical reasons, we lacked CSF control samples without disease, as previously described ^17^. The sample size used was relatively small, which could limit comparison between groups of syndromes, and so the results should be validated using an independent cohort of study participants. Further, in endemic areas, the definition of CM is often challenging and CM is often over-diagnosed. The WHO definition of CM may misclassify up to 25% of cases ^46^ and its specificity is greatly improved by adding a clinical test for Malaria Retinopathy ^47, 48^. However, Retinal changes specific to CM require specialist examination techniques, are difficult to examine in conscious children, and such data was not available in this study. Samples analysed in this study were left over specimens following normal microbiology and chemistry laboratory procedures. There is lacking data for the samples on the time taken from collection during lumbar puncture to storage at -80°C and therefore there is possibility that proteome changes occurred during that period ^49^. Label-free proteomics are cost-efficient, offer higher proteome coverage and a higher dynamic range. However, as every sample is handled separately, variations that can bias the quantitative analysis may be introduced.The results for ABM need to be validated with other aetiologies of ABM and other age groups, such as neonates, since the two pathogens are now uncommon due to the introduction of conjugate vaccines ^50^.

## Conclusions

Children with ABM and CM have different CSF host proteomes ^17^. In the present study, two neutrophil proteins, MPO and LTF, were found to be the best biomarkers to distinguish ABM and CM. They have the potential to be developed as point-of-care diagnostics following validation in an independent cohort. 

## Ethical statement

The samples used in this study had been collected under a previous study examining the mechanisms of neurological damage in Kenyan children with cerebral malaria and acute bacterial meningitis with the approval of KEMRI Scientific Steering Committee (protocol No. 480; by Prof. Charles R. Newton). The parents or guardians of all study participants had provided written informed consent for sample use for future study during sample collection.

## Data availability

The mass spectrometry raw files generated and analysed in the current study have been deposited to the ProteomeXchange Consortium ^51^ ( PXD006357), via the MassIVE partner repository ( MSV000080979), under the following title: Cerebrospinal fluid markers to distinguish bacterial meningitis from cerebral malaria in children.

The FTP for the dataset is available here.

## Supplementary material

**Table S1.** List of quantified proteins.

**Table S2.** List of proteins included in the biomarker analysis quantified proteins.

**Figure S1.** Host response to *Haemophilus influenza* and *Streptococcus pneumoniae* is generic. The principal component analysis score plot depicts overlap of the cerebral spinal fluid proteomes of *H. influenza* and *S. pneumoniae.* HIN, *H. influenza*; SPN, *S. pneumoniae*.

**Figure S2.** A volcano plot showing differential protein expression between acute bacterial meningitis and cerebral malaria, with the latter as the reference. FDR < 0.01 (y axis) and Log FC≥2 (x axis) were set as the significance threshold values as shown with blue lines. FDR, false discovery rate; FC, fold change.

**Figure S3.** Weight assignment of protein biomarkers using the Random Forests mean decrease in accuracy scores. “Important biomarkers” were extracted using a recursive feature elimination (Boruta wrapper package). Each box plot corresponds to mean decrease in accuracy (MDA) scores for 1000 iterations. The proteins are ranked according to their mean importance score. Briefly, MDA scores for actual proteins were compared to those of shadow proteins (randomly shuffled quantities).
