# Supplementary figures and images for: Cerebrospinal fluid markers to distinguish bacterial meningitis from cerebral malaria in children
Source: Wellcome Open Res. 2017 Sep 26;2:47. Originally published 2017 Jul 3. [Version 2] doi: 10.12688/wellcomeopenres.11958.2 (PMC5686508; doi:10.12688/wellcomeopenres.11958.2)

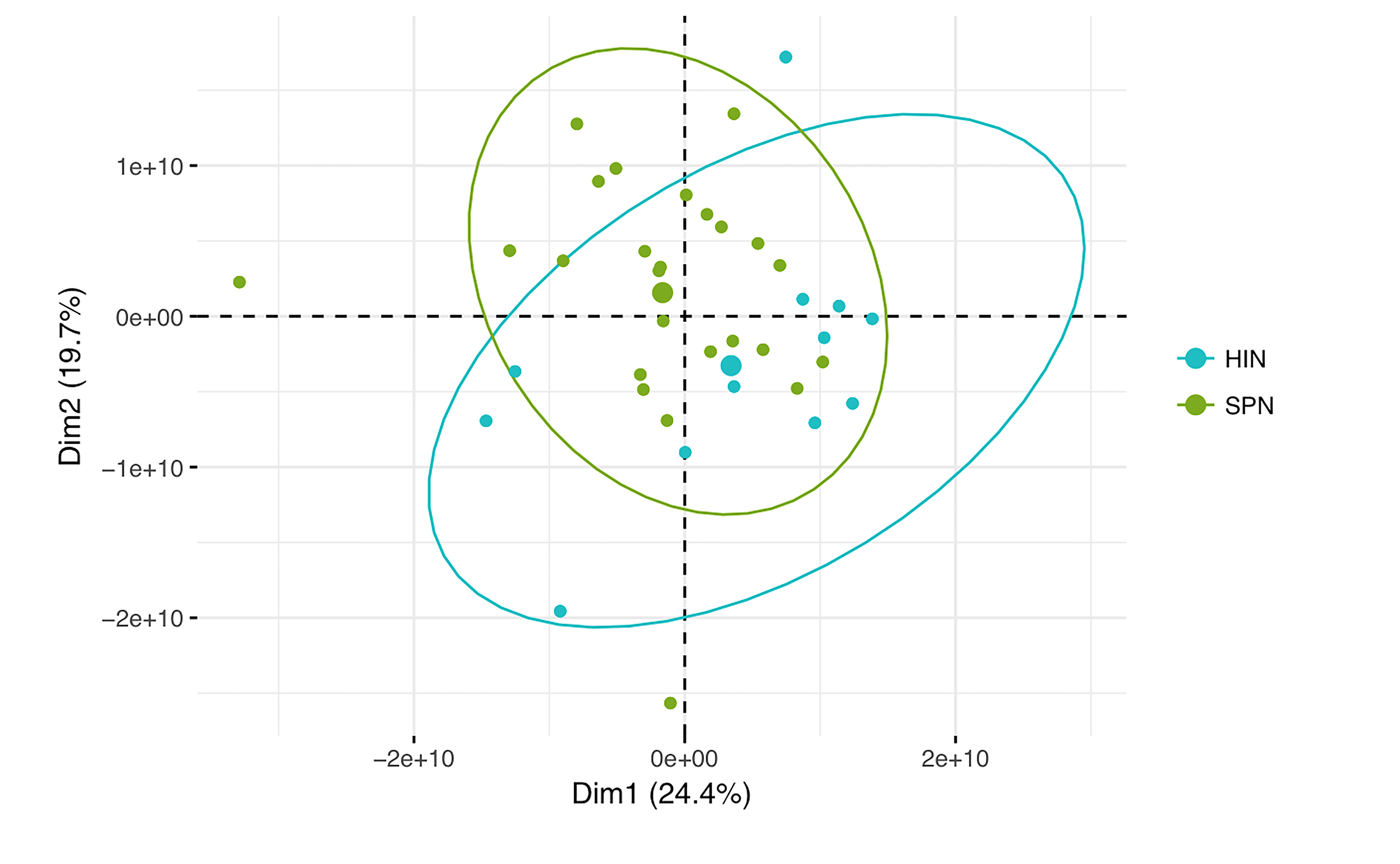

Supplement: Supplementary file 3 [file wellcomeopenres-2-13799-s0003.tgz › c3ed9023-60bc-4aef-b423-f8e8d45d540f.png]

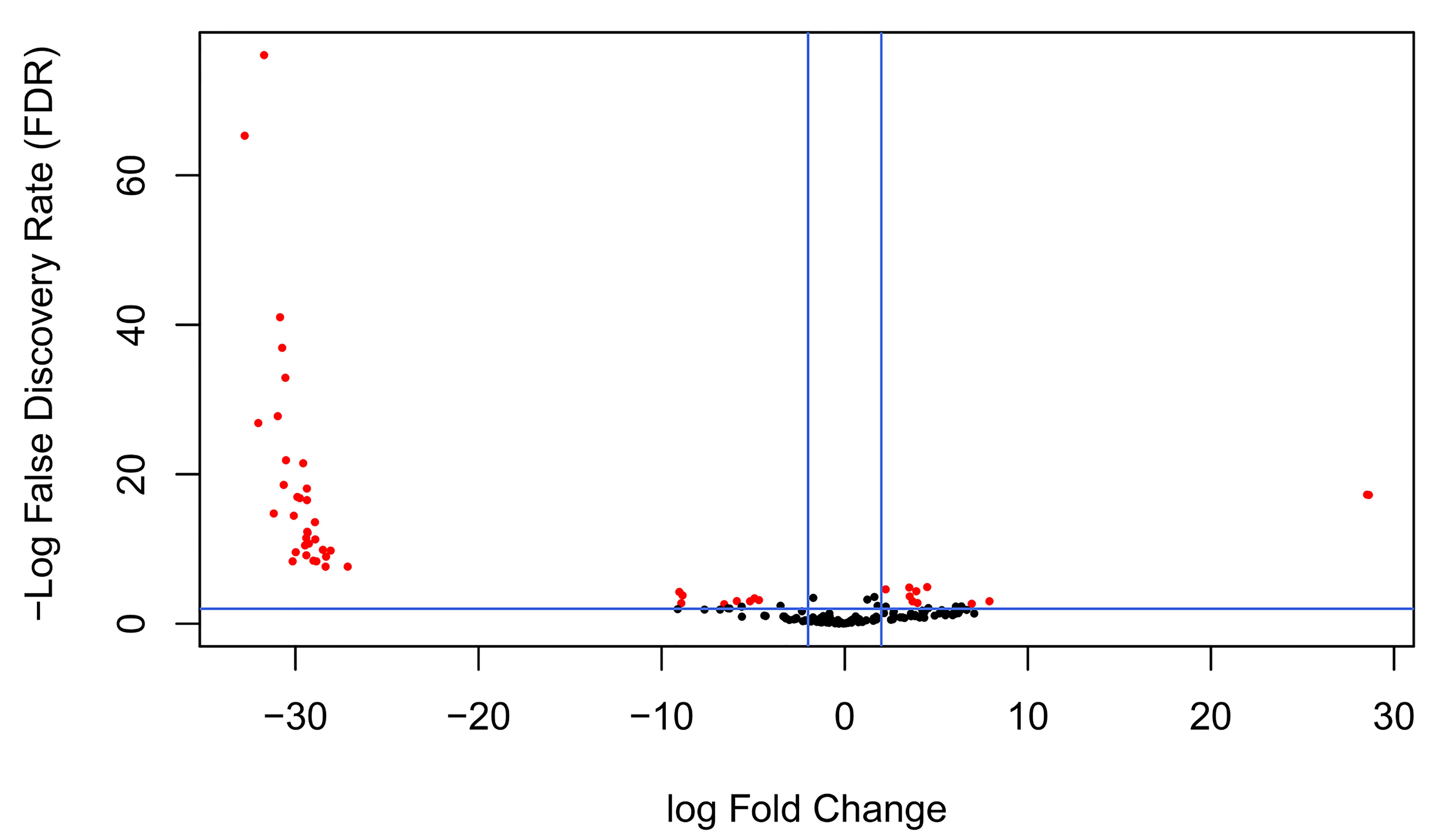

Supplement: Supplementary file 4 [file wellcomeopenres-2-13799-s0001.tgz › 5017d592-8379-4d76-afb7-66c8d9fe21ad.png]

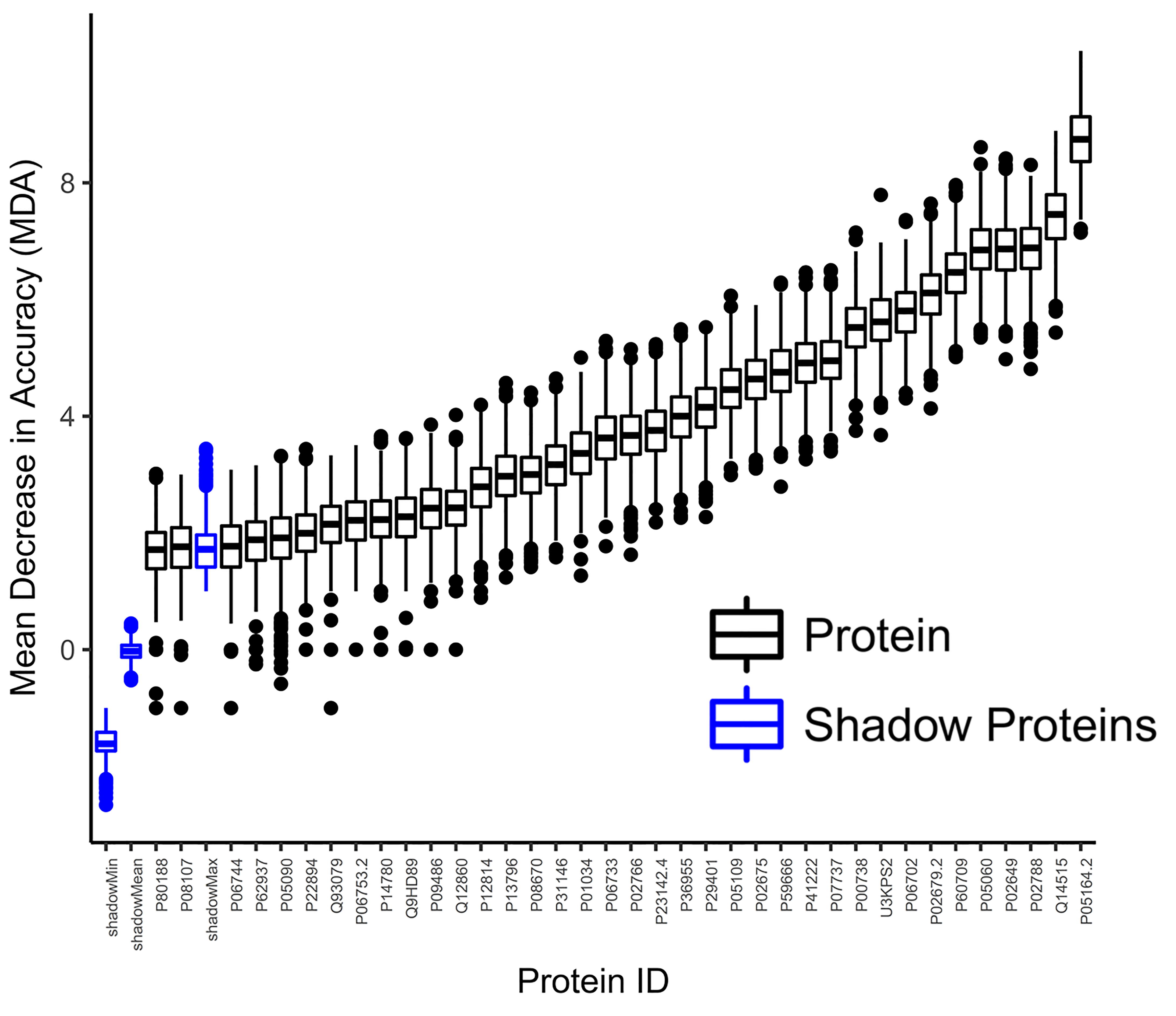

Supplement: Supplementary file 5 [file wellcomeopenres-2-13799-s0002.tgz › d6d1e28a-34b7-43b6-88d0-5cfb8208fab9.png]
