# Supplementary material for: Cerebrospinal fluid markers to distinguish bacterial meningitis from cerebral malaria in children
Source: Wellcome Open Res. 2017 Sep 26;2:47. Originally published 2017 Jul 3. [Version 2] doi: 10.12688/wellcomeopenres.11958.2 (PMC5686508; doi:10.12688/wellcomeopenres.11958.2)
Supplement: Peer review history

# Peer review: Reviewer response for version 2

**DOI:** 10.21956/wellcomeopenres.13799.r26405

**Published:** 2017-11-10

**Authors:** Clarissa Valim

**Affiliations:** 1 Department of Osteopathic Medical Specialties, Michigan State University, East Lansing, MI, USA

The authors response and revisions addressed all my concerns. I have no further comments.

I confirm that I have read this submission and believe that I have an appropriate level of expertise to confirm that it is of an acceptable scientific standard.

# Peer review: Reviewer response for version 2

**DOI:** 10.21956/wellcomeopenres.13799.r26406

**Published:** 2017-09-27

**Authors:** Wilson L. Mandala

**Affiliations:** 1 Malaria Immunology Group, Malawi-Liverpool-Wellcome Trust Clinical Research Programme, Blantyre, Malawi; 2 Academy of Medical Sciences, Malawi University of Science and Technology, Thyolo, Malawi

I am satisfied with the changes made after my initial review. All concerns have been addressed appropriately and satisfactorily. I now approve this manuscript for publication without any reservations.

I confirm that I have read this submission and believe that I have an appropriate level of expertise to confirm that it is of an acceptable scientific standard.

# Peer review: Reviewer response for version 1

**DOI:** 10.21956/wellcomeopenres.12925.r24112

**Published:** 2017-08-14

**Authors:** Clarissa Valim

**Affiliations:** 1 Department of Osteopathic Medical Specialties, Michigan State University, East Lansing, MI, USA

The article by Njunge *et al*. is a very well written report of a sound investigation about biomarkers to differentiate bacterial encephalitis from cerebral malaria in children. The findings of this study, set keystones in the research agenda to develop a so needed point-of-care test for pediatric encephalitis in Africa. We have two major comments and some minor ones.

**Major**

1. The study was conducted with samples collected during 9 years. During these years, were samples from only 59 patients collected and archived? Given the importance of selection bias in biomarker studies, it would be important for the authors to provide details of the criteria used to select patients/samples for this study. Perhaps they could also provide a flowchart of enrollment in the supplementary material.
2. The authors may want to provide minimal details of the collection and storage of their study samples, since Mass Spec proteomics can be very sensitive to details in the collection process including elapsed time to centrifuge and refrigerate samples. If not many details are known, given the age of these samples, it would be helpful to add to the discussion a limitation specifying that some proteins could have been degraded and thus, could not be properly quantified.
  To better understand the importance of selection bias to the author’s results, it would also be helpful to see a distribution of samples by the year they were collected. Hopefully, it can be shown that bacteria samples were collected contemporaneous to malaria samples.
3. The authors state that proteins with more than 50% of samples missing quantification were excluded from the analysis but those that could be quantified in up to 50% of samples a value set to 0 when they could not be quantified. That is fine but it would be helpful to know what was the percent of samples that could not be quantified in each of the two comparison groups for the most important markers (perhaps those included in Table S2). Have the authors considered that an alternative explanation for the non-quantification was that the samples were degraded?

 

**Minor**

  

1. Given the importance of the case classification criteria in this study, the authors may want to provide a few more details of the non-ABM group. They were classified as cerebral malaria but how strong was the evidence of the presence of cerebral malaria in this group? Could they have viral encephalitis, for instance, or a non-detected ABM? Could the authors provide their rational to state that this group represented patients with CM? Regardless of the presence of CM in the group without ABM, the results of the study are relevant since they show biomarkers that identified ABM and non-ABM (or unlikely ABM) patients.
2. The authors may want to include in Table 3, the optimal cut-off value of the proteins that achieve the reported sensitivity, specificity. It would also be helpful for the top 10 or so proteins to see the boxplots comparing the two diagnosis groups to have a better sense of the variability.
3. The authors may want to reconsider and exclude the ROC curve from Figure 4. The curve of an AUC-ROC of approximately 1 is not very illustrative. Also, although the actual accuracy of the identified proteins is probably very high, the AUC-ROC estimated by the authors was probably influenced by the fact that proteins were selected and had their accuracy estimated in the same samples. The curve in this paper is probably giving more weight to the estimated accuracy than it would be necessary.
4. We have a few statistical suggestions for a future study conducted by the authors. However, the authors shall feel free to ignore those suggestions in this study. These different analysis would be unlikely to change conclusions and we believe the results of the study shall be made public and not wait for any additional analysis. The suggestions are:
  a) Wilcoxon and t-tests assuming equal variances were used to compare protein in the two comparison groups. Often the variance of proteins is very different across the two groups and none of these two tests are adequate when the variances vary across comparison groups.
  b) If the number of samples with zeros were substantial, a more appropriate approach would be using Zero inflated or Zero truncated models to compare proteins.
  c) In the future, the authors may want to explore using multiple imputation if missingness is substantial
  d) In the future the authors may want to explore additional feature selection procedures. Random forests are excellent algorithms to create classification signatures but may not be the most appropriate algorithm to select markers. Moreover, conducting a few different algorithms and evaluating consensus may help to decrease a bit the impact of overfitting in each one of these algorithms.
  e) In future studies the authors may explore using re-sampling to estimate accuracy, for instance, using cross-validation. That may help to discount the overestimation in accuracy that occurs when there is neither a validation nor a test set.
5. We could not find any “phenotype” or metadata associated with the protein data. Without that, it would not be possible to reproduce the analysis presented in this paper. I do not consider any action is necessary to address this comment since the authors made available what nearly all investigators do.
6. Alternative explanations for the extremely high estimated AUC-ROC could be inserted in the discussion, such as issues with sample storage and selection that could have differentially affected some proteins (that have different degradation rates), and the impact of misclassification of patients in the CM group.
7. My understanding is that the study used a label-free Mass Spec. To prevent problems with the Mass Spec community, the authors may want to add a comment to the discussion about limitations of the technology used as opposed to a labelled quantitative approach. This is not terribly relevant and it is fine if the authors choose to ignore this comment.

I confirm that I have read this submission and believe that I have an appropriate level of expertise to confirm that it is of an acceptable scientific standard, however I have significant reservations, as outlined above.

# Response

**Published:** 2017-09-21

**Authors:** James Njunge

**Affiliations:** KEMRI-Wellcome Trust, Kenya

Major

1. The study was conducted with samples collected during 9 years. During these years, were samples from only 59 patients collected and archived? Given the importance of selection bias in biomarker studies, it would be important for the authors to provide details of the criteria used to select patients/samples for this study. Perhaps they could also provide a flowchart of enrollment in the supplementary material.

*We provide below bar plots of sample distribution per year. This was a discovery study for biomarkers and we randomly selected samples that had left over CSF samples and fitting study inclusion. We selected samples from a database of approximately 3,420 CSF samples that includes ABM, CM, and unknown aetiology as well as others. Since the proteomics approach used was label free protein quantification, the number of sample that could be used for the discovery study was limited. The study used samples collected over 9 years, although the distribution of these samples for CM was in a span of 4 years (2003 – 2006; Figure 1). For ABM, samples included mainly originated from the years (2002 – 2005 and 2007) (Figure 1.).*

2. The authors may want to provide minimal details of the collection and storage of their study samples, since Mass Spec proteomics can be very sensitive to details in the collection process including elapsed time to centrifuge and refrigerate samples. If not many details are known, given the age of these samples, it would be helpful to add to the discussion a limitation specifying that some proteins could have been degraded and thus, could not be properly quantified.

*In the limitation section, the following statement has been included “Samples analyzed in this study were left over specimens following normal microbiology and chemistry laboratory procedures. There is lacking data for the samples on the time taken from collection during lumbar puncture to storage at -80°C and therefore there is possibility that proteome changes occurred during that period [1]”*

To better understand the importance of selection bias to the author’s results, it would also be helpful to see a distribution of samples by the year they were collected. Hopefully, it can be shown that bacteria samples were collected contemporaneous to malaria samples.

*Figure 1: Yearly distribution of ABM and CM samples included in the study*

3. The authors state that proteins with more than 50% of samples missing quantification were excluded from the analysis but those that could be quantified in up to 50% of samples a value set to 0 when they could not be quantified. That is fine but it would be helpful to know what was the percent of samples that could not be quantified in each of the two comparison groups for the most important markers (perhaps those included in Table S2). Have the authors considered that an alternative explanation for the non-quantification was that the samples were degraded? *The percentages of proteins quantified per group has been provided in Table S2 as requested.*

*In the limitation section, the following statement has been included “Samples analyzed in this study were left over specimens following normal microbiology and chemistry laboratory procedures. There is lacking data for the samples on the time taken from collection during lumbar puncture to storage at -80°C and therefore there is possibility that proteome changes occurred during that period [1]”. * 

Minor

1. Given the importance of the case classification criteria in this study, the authors may want to provide a few more details of the non-ABM group. They were classified as cerebral malaria but how strong was the evidence of the presence of cerebral malaria in this group? Could they have viral encephalitis, for instance, or a non-detected ABM? Could the authors provide their rational to state that this group represented patients with CM? Regardless of the presence of CM in the group without ABM, the results of the study are relevant since they show biomarkers that identified ABM and non-ABM (or unlikely ABM) patients.

*The following text has been included in the limitation section. “Further, in endemic areas, the definition of CM is often challenging and CM is often over-diagnosed. The WHO definition of CM may misclassify up to 25% of cases [2] and its specificity is greatly improved by adding a clinical test for Malaria Retinopathy [3, 4]. However, Retinal changes specific to CM require specialist examination techniques, are difficult to examine in conscious children, and such data was not available in this study.”*

2. The authors may want to include in Table 3, the optimal cut-off value of the proteins that achieve the reported sensitivity, specificity. It would also be helpful for the top 10 or so proteins to see the boxplots comparing the two diagnosis groups to have a better sense of the variability.

Bar plots have been generated as below for review purposes

Figure 2: SPARC

Figure 3: PRTN3

Figure 4: PFN1

Figure 5: MPO

Figure 6: LTF

Figure 7: LCP1

Figure 8: CORO1A

Figure 9: ACTN1

Figure 10: SPARCL1

3. The authors may want to reconsider and exclude the ROC curve from Figure 4. The curve of an AUC-ROC of approximately 1 is not very illustrative. Also, although the actual accuracy of the identified proteins is probably very high, the AUC-ROC estimated by the authors was probably influenced by the fact that proteins were selected and had their accuracy estimated in the same samples. The curve in this paper is probably giving more weight to the estimated accuracy than it would be necessary.

*The AUR-ROC curve has been removed*

4. We have a few statistical suggestions for a future study conducted by the authors. However, the authors shall feel free to ignore those suggestions in this study. These different analysis would be unlikely to change conclusions and we believe the results of the study shall be made public and not wait for any additional analysis. The suggestions are:

a) Wilcoxon and t-tests assuming equal variances were used to compare protein in the two comparison groups. Often the variance of proteins is very different across the two groups and none of these two tests are adequate when the variances vary across comparison groups.

b) If the number of samples with zeros were substantial, a more appropriate approach would be using Zero inflated or Zero truncated models to compare proteins.

c) In the future, the authors may want to explore using multiple imputation if missingness is substantial

d) In the future the authors may want to explore additional feature selection procedures. Random forests are excellent algorithms to create classification signatures but may not be the most appropriate algorithm to select markers. Moreover, conducting a few different algorithms and evaluating consensus may help to decrease a bit the impact of overfitting in each one of these algorithms.

e) In future studies the authors may explore using re-sampling to estimate accuracy, for instance, using cross-validation. That may help to discount the overestimation in accuracy that occurs when there is neither a validation nor a test set.

*We will consider these suggestion which are very appropriate in our biomarker and related work.*

5. We could not find any “phenotype” or metadata associated with the protein data. Without that, it would not be possible to reproduce the analysis presented in this paper. I do not consider any action is necessary to address this comment since the authors made available what nearly all investigators do.

6. Alternative explanations for the extremely high estimated AUC-ROC could be inserted in the discussion, such as issues with sample storage and selection that could have differentially affected some proteins (that have different degradation rates), and the impact of misclassification of patients in the CM group.

*This has been addressed by excluding the AUC-ROC (Figure 4). We have included additional text in the limitation section of the discussion that address challenges (1) with the classification of CM and (2) sample storage.*

7. My understanding is that the study used a label-free Mass Spec. To prevent problems with the Mass Spec community, the authors may want to add a comment to the discussion about limitations of the technology used as opposed to a labelled quantitative approach. This is not terribly relevant and it is fine if the authors choose to ignore this comment.

*We have included the following additional text in the limitation section “Label-free proteomics are cost-efficient, offer higher proteome coverage and a higher dynamic range. However, as every sample is handled separately, variations that can bias the quantitative analysis may be introduced.”*

# Peer review: Reviewer response for version 1

**DOI:** 10.21956/wellcomeopenres.12925.r23986

**Published:** 2017-07-07

**Authors:** Wilson L. Mandala

**Affiliations:** 1 Malaria Immunology Group, Malawi-Liverpool-Wellcome Trust Clinical Research Programme, Blantyre, Malawi; 2 Academy of Medical Sciences, Malawi University of Science and Technology, Thyolo, Malawi

This is a very well written paper which is probably a follow-up to what the group had previously done (Gitao *et al.*, 2013) ^1^. I only have the following reservations;

**Methods section**

1. The study analysed archived CSF samples which had been collected over a period spanning almost nine years. The authors need to say more on how the samples were originally collected, how long it took before they were stored and at what temperature. They may also need to add this as a limitation in the Discussion section bearing in mind that the stability of CSF proteins is compromised if kept under different temperatures for some time (Ranganathan *et al.*, 2006 ^2^).
2. Definition of CM: although archive CSF samples were used, i have strong reservation with the criteria used in defining CM cases. They may need to include this as a limitation and cite recent papers on CM classification in addition to Milner *et al.,* 2014 ^3^ (Beare *et al.*, 2011 ^4^, Severe Malaria, 2014 ^5^, Seydel *et al.*, 2015 ^6^)

**Discussion section**

1. The statement "whilst in CM the blood brain barrier is MILDLY impaired....." may need to be revisited and supported by what has been covered in some recent reviews (Renia *et al.,* 2012 ^7^, Polimeni and Prato, 2014 ^8^, Prato, 2014 ^9^ and what has been published based on ECM in mice models (Poh *et al., *2014 ^10^).)
2. Minor: in Figs 2 and S1, there are some points that seem to overlap (i.e. especially for CM in Fig2). Can these be explained in the Fig legends. For consistency it might also be better to keep the type of labels uniform in all Figs (i.e. if CM is red dot, then it stays that way in all Figs, easier to follow).
3. They may need to double check that in the Results section the number of Tables and Figures being referred to are correct.

I confirm that I have read this submission and believe that I have an appropriate level of expertise to confirm that it is of an acceptable scientific standard, however I have significant reservations, as outlined above.

## References

[1] : Plasma and cerebrospinal proteomes from children with cerebral malaria differ from those of children with other encephalopathies. *J Infect Dis*.2013;208(9) : 10.1093/infdis/jit334 1494-503 10.1093/infdis/jit334 23888081PMC3789566

[2] : Assessment of Protein Stability in Cerebrospinal Fluid Using Surface-Enhanced Laser Desorption/Ionization Time-of-Flight Mass Spectrometry Protein Profiling. *Clin Proteomics*.2006;2(1-2) : 10.1385/CP:2:1:91 91-101 10.1385/CP:2:1:91 20200596PMC2830664

[3] : The systemic pathology of cerebral malaria in African children. *Front Cell Infect Microbiol*.2014;4: 10.3389/fcimb.2014.00104 104 10.3389/fcimb.2014.00104 25191643PMC4139913

[4] : Redefining cerebral malaria by including malaria retinopathy. *Future Microbiol*.2011;6(3) : 10.2217/fmb.11.3 349-55 10.2217/fmb.11.3 21449844PMC3139111

[5] Severe Malaria. *Trop Med Int Health*.2014;19(s1) : 10.1111/tmi.12313_2 7-131 10.1111/tmi.12313_2 25214480

[6] : Brain swelling and death in children with cerebral malaria. *N Engl J Med*.2015;372(12) : 10.1056/NEJMoa1400116 1126-37 10.1056/NEJMoa1400116 25785970PMC4450675

[7] : Cerebral malaria: mysteries at the blood-brain barrier. *virulence*.2012;3(2) : 10.4161/viru.19013 193-201 10.4161/viru.19013 22460644PMC3396698

[8] : Host matrix metalloproteinases in cerebral malaria: new kids on the block against blood-brain barrier integrity?. *Fluids Barriers CNS*.2014;11(1) : 10.1186/2045-8118-11-1 1 10.1186/2045-8118-11-1 24467887PMC3905658

[9] : Blood-Brain Barrier and Cerebral Malaria: The Road So Far. *Journal of Bacteriology & Parasitology*.2014;05(02) : 10.4172/2155-9597.1000e122 10.4172/2155-9597.1000e122

[10] : Damage to the blood-brain barrier during experimental cerebral malaria results from synergistic effects of CD8+ T cells with different specificities. *Infect Immun*.2014;82(11) : 10.1128/IAI.02180-14 4854-64 10.1128/IAI.02180-14 25156726PMC4249313

# Response

**Published:** 2017-09-21

**Authors:** James Njunge

**Affiliations:** KEMRI-Wellcome Trust, Kenya

**Methods section**

1. The study analysed archived CSF samples which had been collected over a period spanning almost nine years. The authors need to say more on how the samples were originally collected, how long it took before they were stored and at what temperature. They may also need to add this as a limitation in the Discussion section bearing in mind that the stability of CSF proteins is compromised if kept under different temperatures for some time (Ranganathan *et al.*, 2006 ^2^).

*The following text has been included in the limitation section. “Samples analyzed in this study were left over specimens following normal microbiology and chemistry laboratory procedures. There is lacking data on the time taken from lumbar puncture to freezing at -80°C for the samples and therefore there is possibility for proteome changes before sample storage.”*******

2. Definition of CM: although archive CSF samples were used, i have strong reservation with the criteria used in defining CM cases. They may need to include this as a limitation and cite recent papers on CM classification in addition to Milner *et al.,* 2014 ^3^ (Beare *et al.*, 2011 ^4^, Severe Malaria, 2014 ^5^, Seydel *et al.*, 2015 ^6^)

*T* *he following text has been included in the limitation section. “Further, in endemic areas, the definition of CM is often challenging and CM is often over-diagnosed. The WHO definition of CM may misclassify up to 25% of cases [2] and its specificity is greatly improved by adding a clinical test for Malaria Retinopathy [3, 4]. However, Retinal changes specific to CM require specialist examination techniques, are difficult to examine in conscious children, and such data was not available in this study.”*

**Discussion section**

1. The statement "whilst in CM the blood brain barrier is MILDLY impaired....." may need to be revisited and supported by what has been covered in some recent reviews (Renia *et al.,* 2012 ^7^, Polimeni and Prato, 2014 ^8^, Prato, 2014 ^9^ and what has been published based on ECM in mice models (Poh *et al., *2014 ^10^).)

*References that support mild impairment to the blood brain barrier during CM compared to bacterial and viral infections have been added as suggested*

2. Minor: in Figs 2 and S1, there are some points that seem to overlap (i.e. especially for CM in Fig2). Can these be explained in the Fig legends. For consistency it might also be better to keep the type of labels uniform in all Figs (i.e. if CM is red dot, then it stays that way in all Figs, easier to follow).

*Overlap of the figures removed in Figure S1 – the CM group has been excluded for clarity*

3. They may need to double check that in the Results section the number of Tables and Figures being referred to are correct.

*This has been thoroughly checked*
